# Evolving trends among *Pseudomonas aeruginosa*: a 12-year retrospective study from the United Arab Emirates

**DOI:** 10.3389/fpubh.2023.1243973

**Published:** 2023-11-30

**Authors:** Jens Thomsen, Godfred A. Menezes, Najiba M. Abdulrazzaq, The UAE AMR Surveillance Consortium, Carole Ayoub Moubareck, Abiola Senok, Dean B. Everett

**Affiliations:** ^1^Department of Occupational and Environmental Health and Safety, Abu Dhabi Public Health Center, Abu Dhabi, United Arab Emirates; ^2^Department of Pathology and Infectious Diseases, Khalifa University, Abu Dhabi, United Arab Emirates; ^3^Department of Medical Microbiology and Immunology, RAK Medical and Health Sciences University, Ras Al Khaimah, United Arab Emirates; ^4^Al Kuwait Hospital Dubai, Emirates Health Services Establishment, Dubai, United Arab Emirates; ^5^College of Natural and Health Sciences, Zayed University, Dubai, United Arab Emirates; ^6^College of Medicine, Mohammed Bin Rashid University of Medicine and Health Sciences, Dubai, United Arab Emirates; ^7^School of Dentistry, Cardiff University, Cardiff, United Kingdom; ^8^Biotechnology Research Center, Khalifa University, Abu Dhabi, United Arab Emirates; ^9^Infection Research Unit, Khalifa University, Abu Dhabi, United Arab Emirates

**Keywords:** *Pseudomonas*, *P. aeruginosa*, multidrug-resistance, national surveillance, healthcare-associated infections, antibiotics, antimicrobial resistance (AMR), UAE

## Abstract

**Introduction:**

*Pseudomonas* is a group of ubiquitous non-fermenting Gram-negative bacteria (NFGNB). Of the several species associated with humans, *Pseudomonas aeruginosa* (PA) can acclimate to diverse environments. The global frequency of PA infections is rising and is complicated by this organism's high intrinsic and acquired resistance to several clinically relevant antibiotics. Data on the epidemiology, levels, and trends of antimicrobial resistance of PA in clinical settings in the MENA/GCC region is scarce.

**Methods:**

A retrospective 12-year analysis of 56,618 non-duplicate diagnostic *Pseudomonas* spp. from the United Arab Emirates (UAE) was conducted. Data was generated at 317 surveillance sites by routine patient care during 2010–2021, collected by trained personnel and reported by participating surveillance sites to the UAE National antimicrobial resistance (AMR) Surveillance program. Data analysis was conducted with WHONET (https://whonet.org/).

**Results:**

Among the total isolates (*N* = 56,618), the majority were PA (95.6%). Data on nationality revealed 44.1% were UAE nationals. Most isolates were from soft tissue (55.7%), followed by respiratory tract (26.7%). PA was more commonly found among inpatients than among outpatients, followed by ICUs. PA showed a horizontal trend for resistance to fluoroquinolones, 3rd- and 4th-generation cephalosporins, and decreasing trends of resistance for aminoglycosides and meropenem. The highest percentage of multidrug resistant (MDR) isolates was reported in 2011 at 35.6%. As an overall trend, the percentage of MDR, extensively drug-resistant (XDR), and possible pandrug-resistant (PDR) isolates generally declined over the study period. Carbapenem-resistant PA (CRPA) were associated with a higher mortality (RR: 2.7), increased admission to ICU (RR: 2.3), and increased length of stay (LOS) (12 excess inpatient days per case), as compared to carbapenem-susceptible PA (CSPA).

**Conclusion:**

The resistance trends in *Pseudomonas* species in the UAE indicated a decline in AMR and in percentages of *Pseudomonas* isolates with MDR and XDR profiles. The sustained *Pseudomonas* spp. circulation particularly in the hospital settings highlights the importance of surveillance techniques, infection control strategies, and stewardship to limit the continued dissemination. This data also shows that CRPA are associated with higher mortality, increased ICU admission rates, and a longer hospitalization, thus higher costs due to increased number of in-hospital and ICU days.

## 1 Introduction

*Pseudomonas* is a group of ubiquitous non-fermenting Gram-negative bacteria (NFGNB) (1). Of the several species associated with humans, *Pseudomonas aeruginosa* (PA) can acclimate to diverse environments due to a varied array of metabolic pathways and inherent pathogenicity due to the existence of several pathogenicity factors and its high genetic flexibility (2). The global frequency of PA infections is rising. This might be attributable in part to the growing incidence of PA infection risk factors, such as an increasing aging population, an increase in long-term illness burden, augmented use of medical devices, and an upsurge in the quantity of immunocompromised persons (1). Enhanced hand hygiene and infection control strategies were projected to reduce the prevalence of several nosocomial infections in hospital settings during the Coronavirus Disease 2019 (COVID-19) pandemic, though this result was not universal (3, 4).

The most common infections caused by PA are respiratory tract infections [such as hospital-acquired pneumonia (HAP) or ventilator-associated pneumonia (VAP)], urinary tract infections (UTI), bloodstream infections (BSI), skin and soft tissue infections, otitis externa and chronic infections in cystic fibrosis (CF) patients (5–7). Lower respiratory tract infections caused by PA have a frequency of 10–20% in VAP (6). The mortality rate in PA*-*VAP and bloodstream infections may be as high as 40% (8). PA infections are exacerbated by this organism's high inherent and acquired resistance to several presently available antibiotics, resulting in increased total healthcare costs and severe, life-threatening disease (9–12).

PA is widely recognized for its ability to resist several antimicrobial agents. According to the World Health Organization (WHO) and the Centers for Disease Prevention and Control (CDC), additional antibiotic research is urgently needed for this one among the priority pathogens, known as the ESKAPE (13, 14). Due to a shortage of treatment choices, clinicians are finding it increasingly challenging to treat PA infections. In 2018, the term “difficult-to-treat resistance” (DTR) was coined. DTR-PA is resistant to a wide range of clinically relevant antibiotics, including ciprofloxacin, levofloxacin, ceftazidime, cefepime, meropenem, aztreonam, piperacillin-tazobactam, and imipenem-cilastatin (4). Resistance mechanisms in PA are classified as intrinsic, acquired, or adaptative. PA is recognized for its intrinsic antibiotic resistance, which is associated with decreased outer membrane (OM) permeability, the formation of efflux pump systems, and the manufacture of antibiotic-inactivating enzymes (15–17). These pathways may be involved in bacterial resistance to β-lactams, quinolones, aminoglycosides, and polymyxins (18, 19). PA has active/overexpressed multidrug efflux pumps, which contribute significantly to antibiotic resistance (9, 20). Aminoglycoside-modifying enzymes and β-lactamases (including penicillinases, cephalosporinases, cephamycinases, and carbapenemases) are produced by PA, capable of selectively inactivating or modifying antibiotics (21–25).

In contrast to intrinsic resistance, acquired resistance is heavily impacted by external factors such as antibiotic exposure (26). Bacterial adaptive resistance is a process that allows bacteria to temporarily strengthen their resistance to the effects of antibiotics or other stresses. Changes in gene and protein expression occur in response to environmental factors. However, when environmental conditions improve, this form of resistance is frequently reversible (9, 17). In PA, these techniques include biofilm production and persister cell growth (27–29).

In a point prevalence study of 28 European countries from 2016 to 2017, PA was the fifth most prevalent cause of hospital-acquired infections (HAI) (30). PA has been found in up to 23% of ICU-acquired infections (31), with resistant PA reaching 48.7% (32). Resistance to piperacillin-tazobactam and meropenem was equivalent in Western Europe and the United States (23%) but greater in Eastern Europe (34.7%) in patients hospitalized with pneumonia between 2019 and 2021 (33). Every year, MDR PA causes 13–19% of HAIs in the United States (34). In Europe, particularly in Greece, MDR and XDR isolates are prevalent (35). A retrospective study of adult hospitalized PA patients in Thailand discovered that XDR strains caused 22% of infections, resulting in significantly higher mortality (36). Another prospective study involving 1,915 ICU patients in India during 2014–2015 found that MDR and XDR strains caused 47.7% of PA infections (37).

MDR PA was most often detected in ICU patients in Saudi Arabia (38). In a 5-year retrospective study conducted in a Saudi multi-hospital healthcare system, meropenem and ceftazidime had the lowest (82–83%) susceptibility (39). Previous studies reported 15% carbapenem resistance in PA in Oman (40) and 41% in Lebanon (41). In a retrospective study conducted in Tawam hospital, Al Ain, from 2004 to 2008, PA showed significant reductions in sensitivity to almost all the antibiotics tested (42). In Dubai hospitals, 23.9% of PA are carbapenem-resistant and most of the strains are part of a large clone, showing clonal dispersion (43).

Treatment options for a suspected/confirmed susceptible PA strain should be conservative, saving newer antibiotics and picking the optimum alternative for MDR/XDR isolates depending on particular resistance mechanisms. Cefiderocol and imipenem-cilastatin-relebactam due to broad antibacterial activity, including against carbapenem-resistant may remain active in situations when other new antibiotics have failed (44).

The increased threat posed by AMR infections extends beyond developing nations to the Middle East and North Africa (MENA) area. The United Arab Emirates (UAE) is a country in the Gulf Cooperation Council (GCC) region recognized for its cosmopolitan atmosphere, diversity of races and cultures, and rising prominence as an international travel, tourist, financial, and health sector hub. The spread of resistant pathogens is facilitated by a diverse and heterogeneous population, and PA is no exception. Nonetheless, there has never been thorough, long-term research of the evolution and variations in PA resistance traits in the UAE. Because PA species are nosocomial and robust, longitudinal, retrospective surveillance studies of such infections in the UAE are necessary. The objective of the current study is to describe the longitudinal changes in the nationwide antimicrobial resistance aspects of PA spanning all seven emirates of the UAE. It represents the first documentation of antimicrobial resistance in PA isolated from UAE medical centers over a period of 12 years, from 2010 to 2021.

## 2 Materials and methods

### 2.1 Study design and data source

Data were generated and cleaned through the UAE national AMR Surveillance programs as described by Thomsen et al. (45, 46). The UAE national AMR surveillance program has adopted the Global AMR Surveillance System protocol (GLASS, World Health Organization). Participation of surveillance sites and laboratories, as well as nomination of AMR surveillance focal points is the initiative of each individual site. A multi-institutional retrospective observational study was conducted between 2010 and 2021 in the UAE using data gathered through the national WHONET microbiology laboratory database software (http://www.whonet.org).

### 2.2 Identification and enrollment of national AMR surveillance sites

In 2010, AMR surveillance was established by the Department of Health Abu Dhabi (DoH) in the Emirate of Abu Dhabi. In 2014, the Ministry of Health and Prevention established AMR surveillance at the national level. As such, starting 2010, UAE institutions were gradually enrolled into the UAE national AMR surveillance program, and enrollment was based on epidemiological needs assessment, readiness, and willingness of facilities to participate, availability of high-quality electronic AMR data, lab accreditation status, and qualification and training of staff. Since 2014, hospitals, centers, and clinics were representing all seven emirates of the UAE (45, 46).

### 2.3 Bacterial population and variables of the study

All *Pseudomonas* spp. isolated from clinical samples by medical professionals in the National AMR surveillance sites were included in this study from January 2010 to December 2021. Only the first isolate per patient, species and reporting period was included in the surveillance analysis. Not included were quality control isolates, screening isolates, duplicate isolates, non-diagnostic isolates (e.g., infection control isolates, environmental isolates), and isolates from primary contaminated sources (pedibag).

The associated patient demographic, clinical, and microbiologic data of laboratory test results were extracted. The demographic variables included age, sex, nationality, patient location (e.g., ward, clinic), patient location type (e.g., inpatient, outpatient, ICU), facility type reporting the isolate (hospital/center/clinic). Clinical variables included discharge health outcome (death/alive), and microbiology variables included specimen collection date, specimen source, organism name, antibiotic name, and antibiotic susceptibility testing results. The infection was considered community acquired if it originated outside the clinical environment in cases of outpatients or patients presenting with the infection at the emergency department. The infection was considered nosocomial if the infection was identified in an inpatient setting such as critical (ICU) or non-critical (non-ICU) care environment. The U.S. Centers for Disease Prevention and Control (CDC) definitions for hospital-acquired and community acquired definitions (HAI/CAI) could not be strictly applied as case-based clinical data for establishing a diagnosis of HAI/CAI was not routinely available.

### 2.4 Bacterial identification

Bacterial identification was performed at the national AMR surveillance sites by medical professionals. The participating centers used at least one commercial, automated system for identification of bacteria, including VITEK^®^ (BioMérieux SA, Craponne, France), BD Phoenix™ (Becton Dickinson, New Jersey, USA) and MicroScan WalkAway (Beckman Coulter, Brea, CA, USA). Only one lab relied on manual systems like API^®^ (Analytical Profile Index. BioMérieux SA, Craponne, France) solely for identification. Unusual test results were confirmed locally.

### 2.5 Antimicrobial susceptibility testing

Antimicrobial susceptibility testing was performed at the National AMR surveillance sites using at least one commercial, automated system for routine antimicrobial susceptibility testing. Only two laboratories used manual testing methods (disc diffusion/Kirby Bauer). The antibiotics that were tested for susceptibility were selected as per clinical requirements for routine patient care by the participating surveillance laboratories. All labs followed CLSI guidelines for antimicrobial susceptibility testing of bacteria (CLSI-M100) (47). To assess the MDR phenotype of the isolates, as well as the possibly extensively drug-resistant (XDR) and possibly pandrug-resistant (PDR) phenotypes, a modified version of the standard definition by Magiorakos et al. was used (12). Magiorakos' et al. definitions for XDR and PDR phenotypes for *Pseudomonas* spp. include 8 antimicrobial categories with 17 antibiotic agents. For technical reasons, associated costs, and local formulary requirements, participating laboratories would not routinely test all 17 antibiotics, i.e., some antibiotics were only very rarely or not at all tested. As such, the following, slightly modified definitions were used for “possible XDR” and “possible PDR” isolates (modifications highlighted in *italics*):

- **“Possible XDR”**: Non-susceptibility to at least one agent *routinely tested by clinical labs* in all but two or fewer antimicrobial categories (i.e., bacterial isolates remain susceptible to only one or two categories).
- **“Possible PDR”**: Non-susceptibility to all agents *routinely tested by clinical labs* in all antimicrobial categories (i.e., no agents were tested as susceptible for that organism).

Multidrug-resistant (MDR) bacteria are those that are non-susceptible to at least one agent in three or more antibiotic classes; extensively drug-resistant (XDR) bacteria are those that are non-susceptible to at least one agent in all categories but susceptible to two or less antimicrobial categories; and pan-drug-resistant (PDR) bacteria are those that are non-susceptible to all agents in all antimicrobial categories (12).

### 2.6 Statistical analysis

We determined the annual trends of antimicrobial resistance if data were available for at least five consecutive years. Where fewer than 30 isolates per year were reported, or data was not available for all the years within the study period, trend analysis for antimicrobial resistance was not conducted. We assessed the statistical significance of trends of antimicrobial resistance by using a Chi-square for trend test (extended Mantel-Haenszel). This was computed using Statistical Package for the Social Sciences (SPSS) v29 software package. EpiInfo^TM^ (https://www.cdc.gov/epiinfo/index.html) was used for assessing the significance of difference in mortality rates and ICU admission rates, for which a Chi^2^-test of independence was applied. To evaluate differences in length of stay (LOS) between patients with CSPA and patients with CRPA, we performed a weighted log -rank test. For all statistical analysis tests a *p*-value of *p* < 0.05 was considered significant.

## 3 Results

### 3.1 Distribution of reporting sites for national AMR surveillance

**Figure 1:**
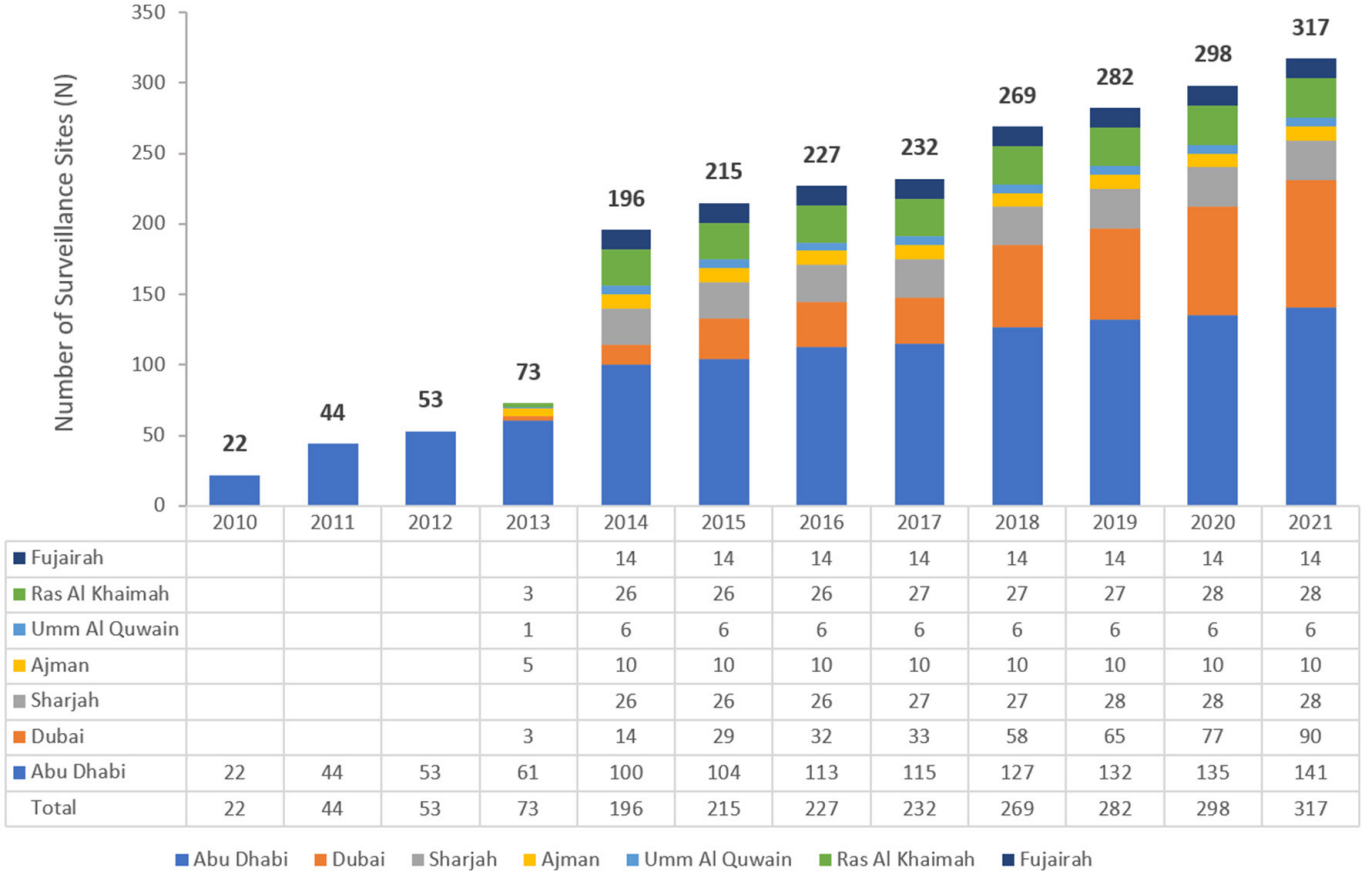
The number of participating sites over the years of the surveillance period—by Emirate (UAE, 2010–2021).

AMR surveillance in the United Arab Emirates was initiated in 2010 in the Emirate of Abu Dhabi by the Health Authority Abu Dhabi, with six hospitals and 16 centers/clinics enrolled. In 2014, the Ministry of Health and Prevention (MOHAP) initiated AMR surveillance on the national level. Additional sites were enrolled over the years, starting with the 22 participating sites located only in the Emirate of Abu Dhabi in 2010, which is the first year during which the study was initiated, and reaching in 2021 a total of 317 surveillance sites, including 87 hospitals and 230 centers/clinics and representing all seven emirates of the country. Figure 1 represents the distribution of reporting sites by Emirate from 2010 to 2021. It is worth mentioning that the Emirate of Abu Dhabi had the highest number of contributing sites, namely 141 (44.2%) out of the total 317 sites enrolled in this study.

### 3.2 Bacterial population

**Figure 2:**
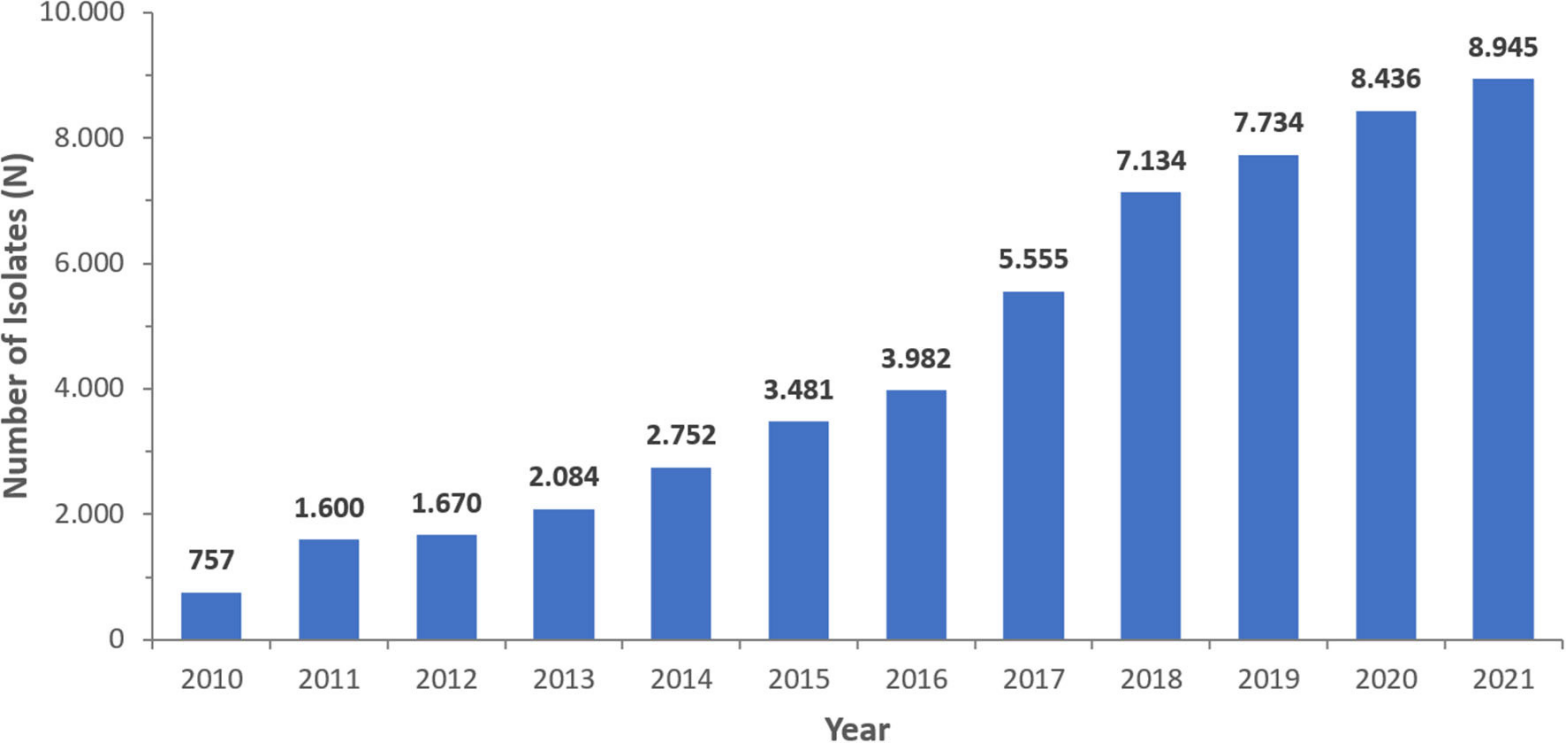
Number of *Pseudomonas aeruginosa* isolates/patients reported per year (UAE, 2010–2021), *N* = 54,130.

From 2010 to 2021, a total of 56,618 non-repetitive *Pseudomonas* spp. were isolated from an equivalent number of patients over the surveillance period. The number of isolates increased from 770 in 2010 to 9,699 in 2021, corresponding with the increasing number of reporting sites (2010: *n* = 22; 2021: *n* = 317). A steady increase in the number of reported isolates occurred, particularly in 2020 (*n* = 8,783) and 2021 (*n* = 9,699) during the COVID-19 pandemic. As shown in Table 1, among the total isolates reported, the majority were PA (95.6%). Figure 2 represents the number of *Pseudomonas* spp. included per year. Since the number of reporting sites was increasing over the study period (2010–2021), the number of reported isolates was increasing as well.

**Table 1:** Number of *Pseudomonas* spp. reported (2010–2021), *N* = 56,618.

**Sl. No**.	**Organism**	**Number of isolates**	**(%)**
1	*Pseudomonas aeruginosa*	54,130	95.61
2	*Pseudomonas* sp.	908	1.61
3	*Pseudomonas putida*	782	1.40
4	*Pseudomonas stutzeri*	391	0.70
5	*Pseudomonas fluorescens*	299	0.53
6	*Pseudomonas mendocina*	58	0.10
7	*Pseudomonas pseudoalcaligenes*	25	0.04
8	*Pseudomonas alcaligenes*	24	0.04
9	*Pseudomonas anguilliseptica*	1	0.02
**Total**		**56,618**	100

### 3.3 Distribution of *P. aeruginosa* patients by gender, age, nationality status, and hospital location

**Figure 3:**
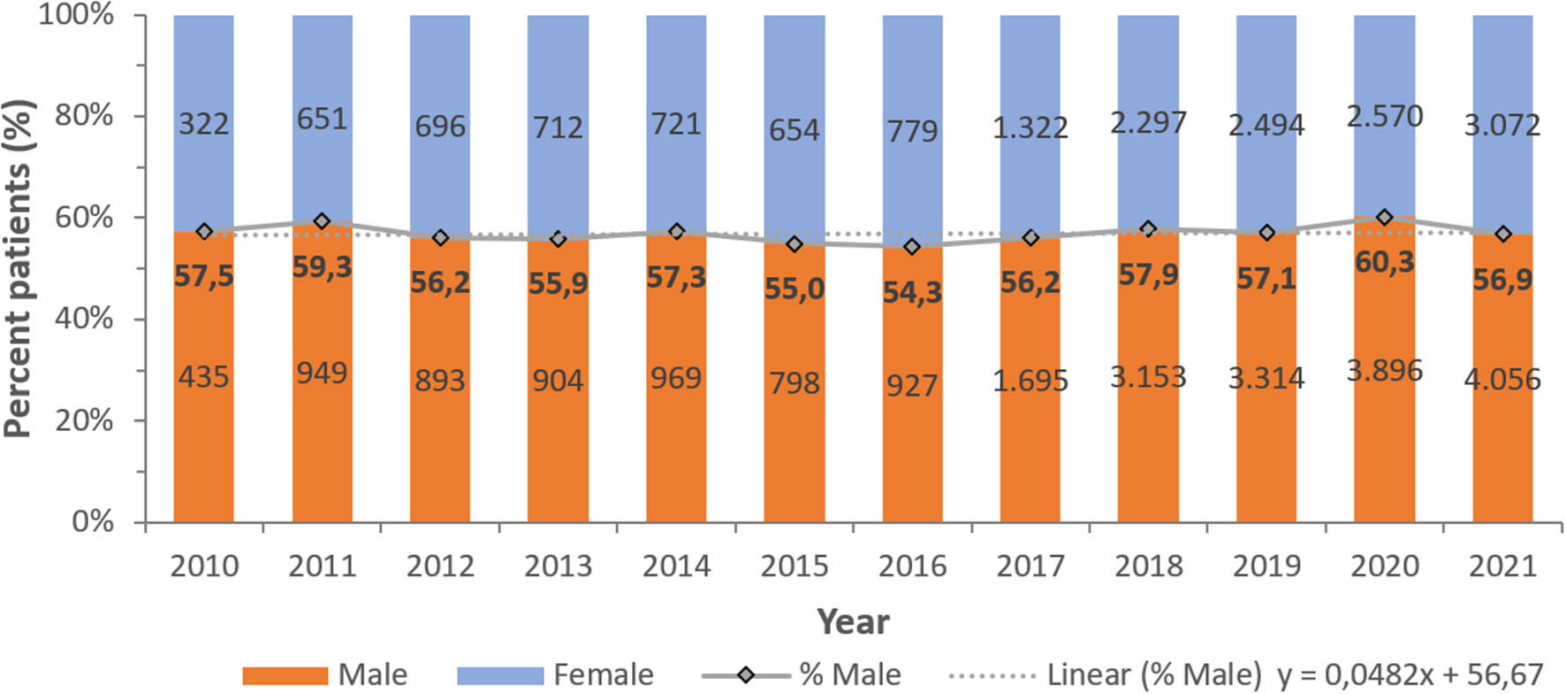
Distribution of *Pseudomonas aeruginosa* patients per year—by gender (UAE, 2010–2021), *N* = 54,130.

**Figure 4:**
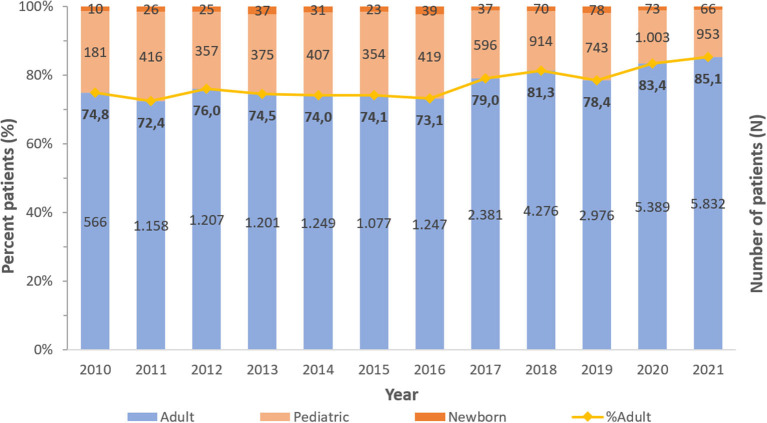
Distribution of *Pseudomonas aeruginosa* patients per year—by age group (UAE, 2010–2021), *N* = 54,130.

**Figure 5:**
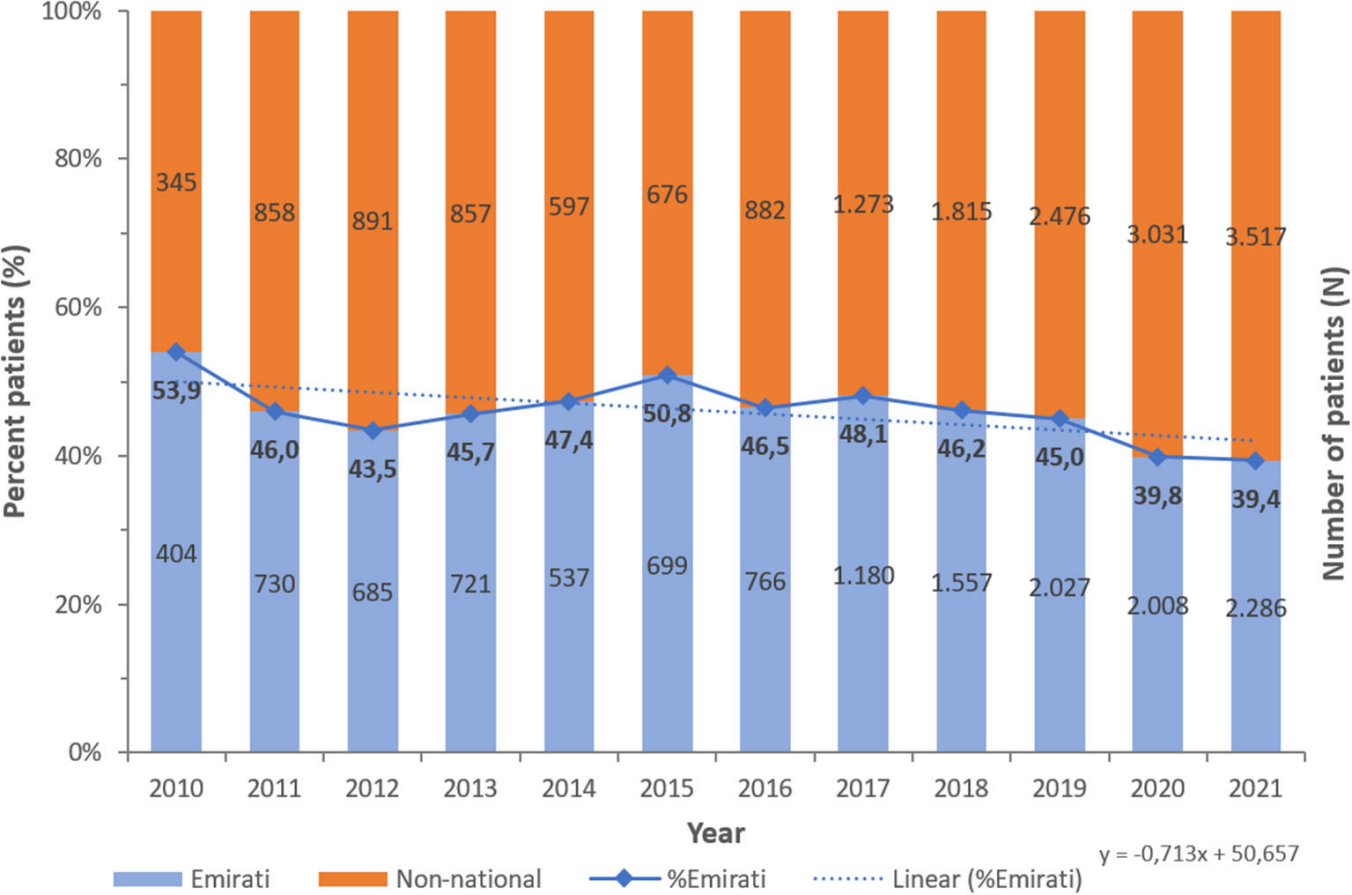
Number of *Pseudomonas aeruginosa* per year—by Nationality status (UAE, 2010–2021), *N* = 54,130 [13,600 (44.1%) Emiratis; 17,218 (55.9%) non-national].

A consistent preponderance of males and adult patients was observed (Table 2; Figures 3, 4). PA were mostly associated with adults (79.8%), as compared to children and newborns (20.2%). Data on nationality was available for 30,818 patients of whom 44.1% were UAE nationals while the remaining 55.9% of patients comprised of expatriate individuals from across 144 nationalities (Figure 5). There was a decreasing trend of percentage Emiratis with PA, which was statistically highly significant (*p* < 0.001). Most patients were detected in the Emirate of Abu Dhabi (29,721; 54.9%), followed by Dubai (12,606; 23.3%), Sharjah (3,989; 7.4%), Ras al Khaimah (3,533; 6.5%), Umm Al Quwain (1,714; 3.1%), Ajman (1,390; 2.6%), and Fujairah (1,177; 2.2%).

**Table 2:** Demographic distribution of patients with *Pseudomonas aeruginosa* −2010–2021.

**Demographics**	**Number of patients (N** = **54,130)**		**Percentage**
Gender	Male	21,989	40.62%
	Female	16,290	30.10%
	Unknown	15,851	29.28%
Age group	Newborn	515	0.95%
	Pediatric	6,718	12.41%
	Adult	28,559	52.76%
	Unknown	18,338	33.88%
Nationality	Emirati	13,600	25.12%
	Non-Emirati	17,218	31.81%
	Unknown	23,312	43.07%
Hospital location	Outpatient	18,166	33.56%
	Inpatient (excluding ICUs)	16,847	31.12%
	Intensive Care Unit	6,223	11.50%
	Others	12,894	23.82%

Definitions: Newborn = 0–30 days; Pediatric = between 30 days and 19 years; Adult = >19 years.

### 3.4 Distribution of *P. aeruginosa* by specimen type group

Out of *N* = 54,130 non-duplicate PA isolates, most isolates were from soft tissue (45.7%), respiratory tract (26.7%), and urine (19.8%), followed by blood (2.8%), genital (0.8%), stool (0.3%), cerebrospinal fluid (0.05%), and others (3.8%).

### 3.5 Distribution of *P. aeruginosa* by location (inpatient/outpatient) and department

PA was more commonly found among inpatients (42.6%, including ICU: 11.5%) than among outpatients (33.6%). The majority of the patients were enrolled in medical (20.4%) and surgical (16.4%) departments.

### 3.6 Antimicrobial resistance trends for *P. aeruginosa*

**Figure 6:**
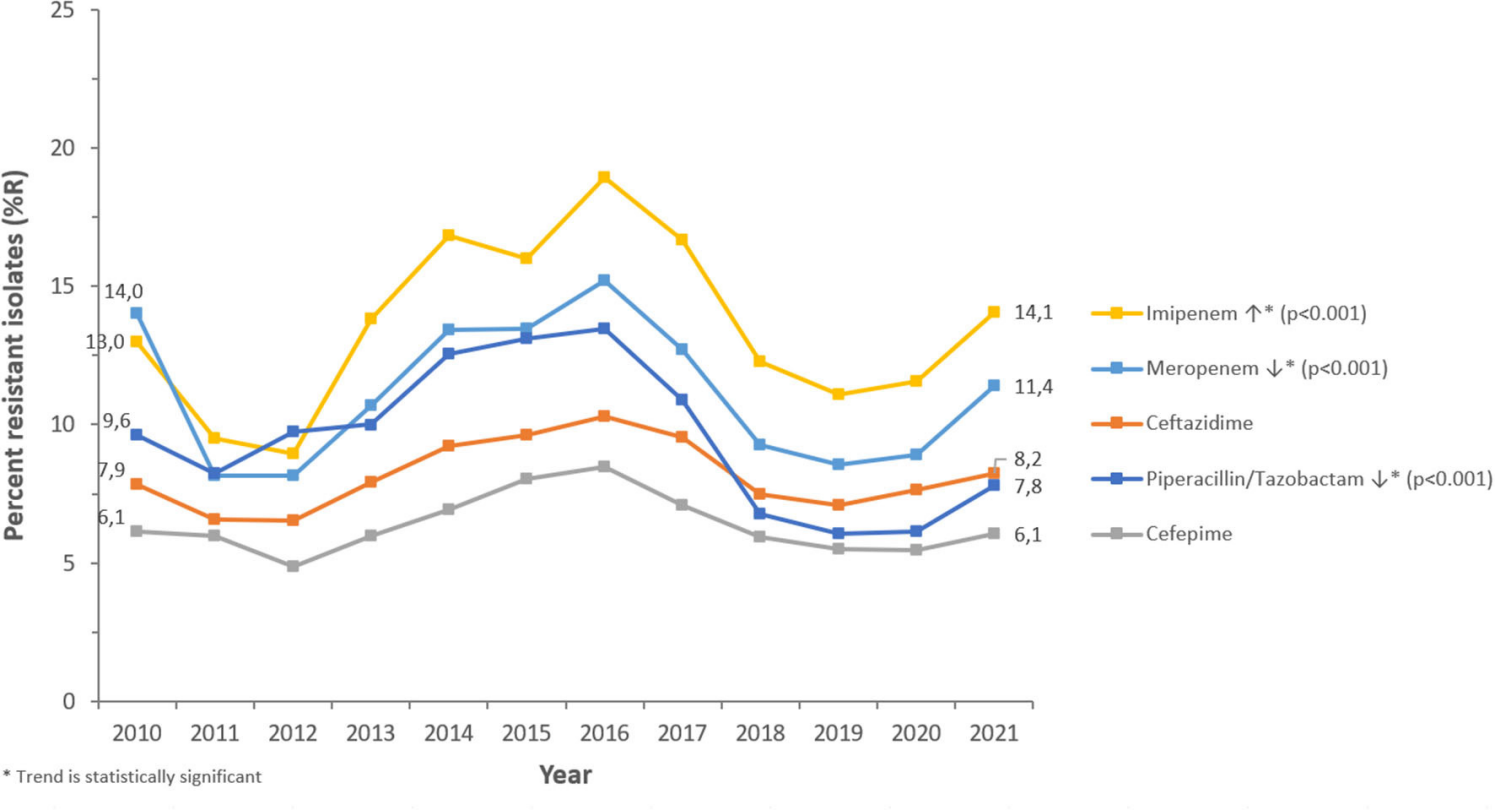
Resistance trends of *Pseudomonas aeruginosa* for beta-lactam antibiotics (UAE, 2010–2021).

**Figure 7:**
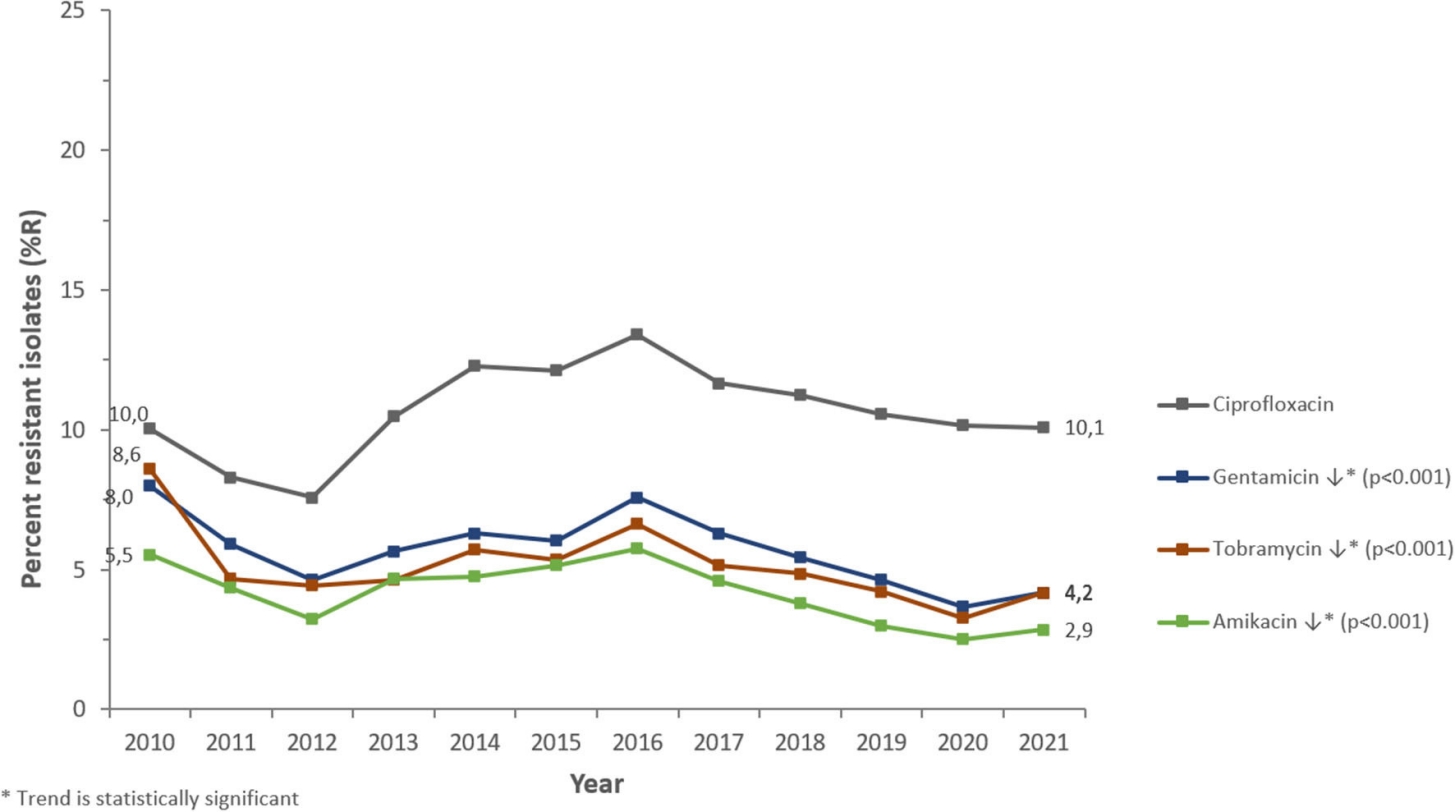
Resistance trends of *Pseudomonas aeruginosa* for non-beta-lactam antibiotics (UAE, 2010–2021).

PA was most frequently tested for aminoglycosides (98.6% of PA isolates), fluoroquinolones (98.5%), 3rd- and 4th-generation cephalosporins (98.1%/94.6%), carbapenems (94.5%), and beta-lactam/beta-lactamase-inhibitor (BL/BLI)-combinations (89.4%), among other antibiotics (>50 antibiotics in total). PA showed increasing or decreasing long-term trends of resistance (%R) for several antibiotics during the study period (Figures 6, 7).

Among the beta-lactam antibiotics, resistance was highest for carbapenems (2021: imipenem 14.1 %R, meropenem 11.4 %R), whereas piperacillin/tazobactam, ceftazidime, and cefepime showed resistance levels of 7.8, 8.2, and 6.1%R, respectively (2021). Among non-beta-lactam antibiotics, the highest resistance was observed for ciprofloxacin (2021: 10.1%R). Resistance to aminoglycosides (amikacin, gentamicin, tobramycin) was 2.9, 4.2, and 4.2%R (2021), respectively.

PA showed a decreasing trend of resistance to broad-spectrum penicillins (piperacillin-tazobactam: from 9.6%R in 2010 to 7.8%R in 2021) (*p* < 0.001) and overall horizontal trends for resistance to 3rd- and 4th-gen. cephalosporins (ceftazidime, cefepime). Resistance trends for carbapenems were diverse: imipenem showed a slightly increasing long-term trend of resistance, from 13.0%R (2010) to 14.1%R (2021) (*p* < 0.001), whereas meropenem showed a decreasing long-term trend of resistance, from 14.0%R (2010) to 11.4%R (2021) (*p* < 0.001). PA showed an overall horizontal trend of resistance for fluoroquinolones (ciprofloxacin) and decreasing trends of resistance for aminoglycosides (gentamicin, amikacin, tobramycin). From a short-term perspective, it is noteworthy that resistance levels were decreasing for all antibiotics studied during the period 2010 to 2012, then increasing for all antibiotics during the period 2012 to 2016 (reaching an all-time high in 2016), then again decreasing during the period 2017–2019 (beta-lactam antibiotics) or 2020 (non-beta-lactam antibiotics), and since then increasing again until 2021.

**Figure 8:**
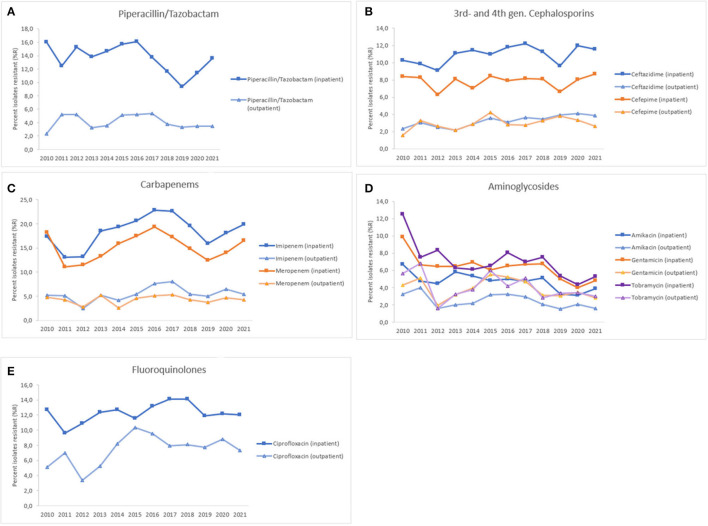
Resistance trends of *Pseudomonas aeruginosa*, inpatient vs. outpatient, United Arab Emirates, 2010–2021. **(A)** Piperacillin/Tazobactam, **(B)** 3rd- and 4th-gen. cephalosporins, **(C)** Carbapenems, **(D)** Aminoglycosides, **(E)** Fluoroquinolones.

A sub-group analysis investigated antimicrobial resistance trends of inpatients (ICU, non-ICU), as compared to outpatients (outpatient and emergency departments, centers, clinics) (Figure 8). Generally, resistance levels were higher for inpatients as compared to outpatients. For piperacillin/tazobactam, PA showed resistance levels between 9.4 and 16.1%R for inpatients, whereas resistance levels were between 2.4 and 5.3%R for outpatients, both with a decreasing trend over time (inpatient: *p* < 0.001, outpatient: n.s.). For 3rd-gen. cephalosporins (ceftazidime), resistance levels were between 9.1 and 12.2%R (inpatients), and between 2.1 and 4.1%R (outpatients), both showing increasing trends (inpatients: n.s., outpatients: *p* < 0.01). For 4th-gen. cephalosporins (cefepime), resistance levels were between 6.3 and 8.7%R (inpatients), and between 1.6 and 4.2%R (outpatients), both showing increasing trends (inpatients: n.s., outpatients: n.s). For carbapenems (imipenem, meropenem), PA showed resistance levels between 11.1 and 22.9%R for inpatients, whereas resistance levels were between 2.5 and 8.0%R for outpatients. Both, imipenem and meropenem showed a slightly increasing trend of resistance (%R) for inpatients and outpatients during 2010–2021, however these trends were statistically not significant. For aminoglycosides (amikacin, gentamicin, tobramycin), resistance levels were between 3.1 and 12.5%R (inpatients), and between 1.5 and 6.8%R (outpatients), both showing decreasing trends (inpatients: *p* < 0.001, outpatients: *p* < 0.05). For fluoroquinolones (ciprofloxacin), PA showed resistance levels between 9.6 and 14.1%R for inpatients, whereas resistance levels were between 3.3 and 10.4%R for outpatients, both with an increasing trend over time (inpatients: n.s., outpatients: *p* < 0.05) (Figure 8).

**Figure 9:**
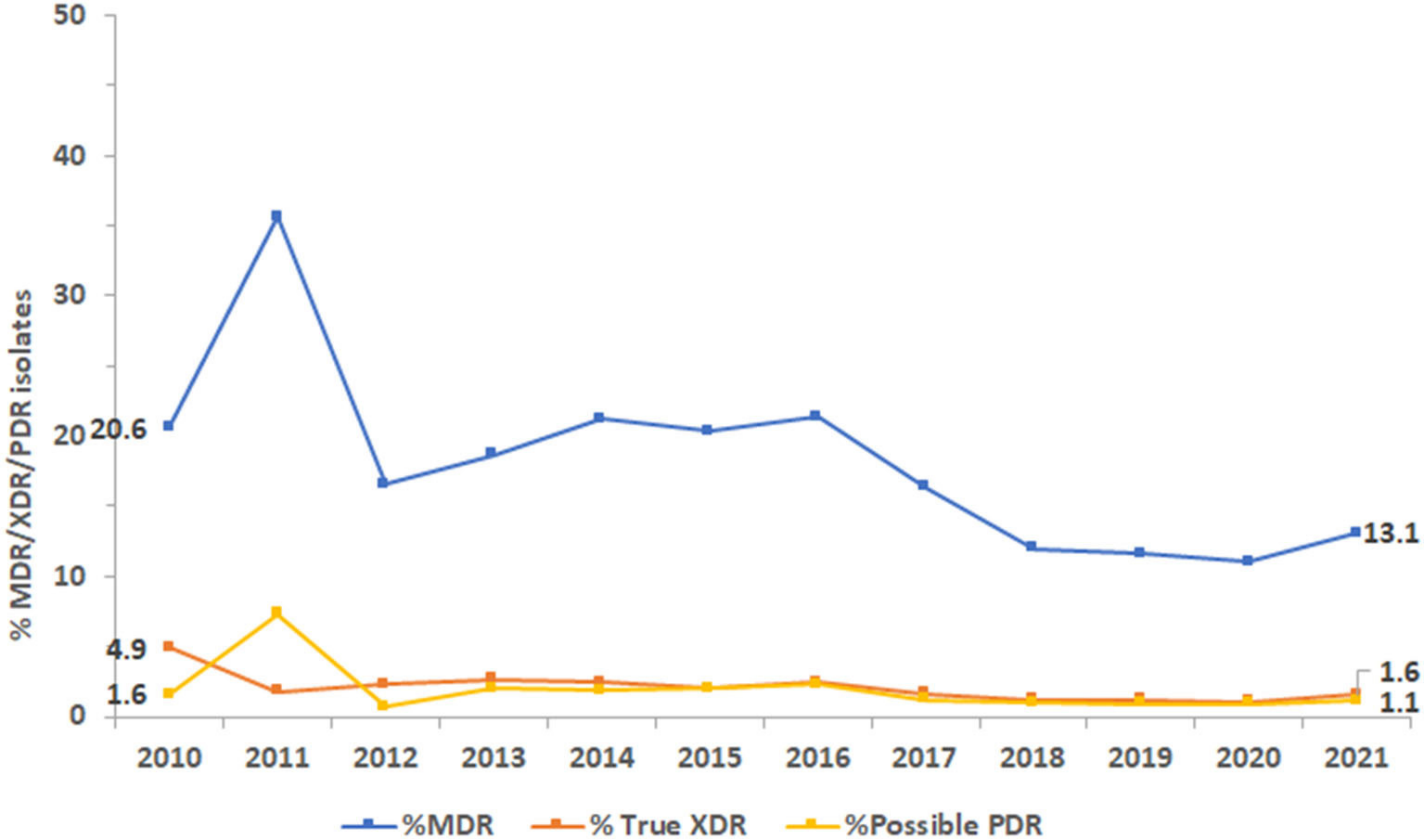
Trends of *Pseudomonas aeruginosa* isolates for multidrug-resistant (MDR), extensively drug-resistant (XDR), and possible pandrug-resistant (PDR) phenotypes over the study period.

The proportion of isolates with MDR phenotype, as shown in Figure 9, being resistant to three or more classes of antibiotics was 20.6% in 2010 and 13.1% in 2021. The highest percentage of MDR isolates was reported in 2011 at 35.6%. Similarly, the highest percentage of XDR isolates was reported in 2010 at 4.9% and of possible PDR isolates in 2011 at 7.3%. As an overall trend, the percentage of MDR, XDR, and possible PDR isolates generally declined over the study period especially starting from the year 2016, as shown in Figure 9, although a slight increase has been observed for %MDR between 2020 and 2021.

### 3.7 Mortality rate

A subgroup analysis including the nine clinical institutions that reported mortality health outcome data was performed. In these institutions, a total of 10,090 patients were associated with carbapenem-susceptible *P. aeruginosa* (CSPA) of whom 706 patients died (mortality rate: 7.0%), while a total of 1,492 patients were associated with carbapenem-resistant *P. aeruginosa* (CRPA), of whom 281 patients died (mortality rate: 18.8%). The difference in mortality between CRPA patients (18.8%) and CSPA patients (7.0%) is statistically highly significant [RR = 2.6917 (2.3704, 3.0565), Chi-square = 233.6096, *p* < 0.001]. CRPA patients were 2.69 times more likely to be discharged as “expired” (i.e., dead), as compared to CSPA patients.

### 3.8 Admission to intensive care unit

A total of 35,563 patients were associated with CSPA of whom 4,521 patients were admitted to ICU (ICU admission rate is 12.71%), while a total of 5,724 patients were associated with CRPA, of whom 1,702 patients were admitted to ICU (ICU admission rate: 29.73%). The difference in ICU admission rate is statistically highly significant [RR = 2.3390 (2.2288, 2.4546), Chi-square = 114.6626, *p* < 0.001]. CRPA patients were 2.34 times more likely to be admitted to ICU, as compared to CSPA patients.

### 3.9 Length of stay

A subgroup analysis was conducted for inpatients for whom the date of admission as well as the date of discharge from hospital were known, i.e., the duration of hospitalization (length of stay, LOS) could be calculated. For 3,521 patients associated with CSPA, the median length of stay was 8 days, as compared to patients who were associated with CRPA (*n* = 556), whose median length of stay was 20 days (Supplementary Figure 1). The difference in LOS was also visualized using Kaplan-Meier curves (Supplementary Figure 2). To assess if the observed difference in length of stay (LOS) was statistically significant, we performed a weighted log-rank test since group sizes were different and we gave more weight to the smaller group so as to make them comparable. The chi square test was 129, with a *p* < 0.0001, showing that the difference in hospitalization duration (length of stay/LOS) between patients who were associated with CSPA and those who were associated with CRPA was statistically significant.

Based on a total of *n* = 7,607 patients associated with CRPA during the observation period (2010–2021), a total of 91,284 excess days of hospitalization were observed, attributable to CRPA. For the year 2021 only (*n* = 1,296 CRPA cases), a total of 15,552 excess hospitalization days were observed, attributable to CRPA.

## 4 Discussion

PA is a common, facultative pathogenic bacterium associated with HAIs and the global frequency of PA infections is rising (1). The emergence and dissemination of MDR and XDR PA are serious public health concerns worldwide. MDR PA infections have increased globally, accounting for up to 30% of PA infections in some regions (48). This is the first comprehensive study in the UAE to show the nationwide prevalence of *Pseudomonas* in clinical settings, as well as changes in antibiotic resistance patterns. The current study used a vast dataset accumulated over a relatively long period of time, allowing for comprehensive monitoring of even subtle variations in antibiotic resistance among *Pseudomonas*. This kind of in-depth analysis has never been carried out before in the country. The non-repetitive *Pseudomonas* samples studied in this study have laboratory-confirmed identity and antibiotic resistance profiles, demonstrating the validity of the microbiological material used and the accuracy of the data gathered. The finding of a decline in antibiotic resistance in *Pseudomonas* over around 12 years is perhaps the most thought-provoking finding in this study, and this was observed despite an increase in the number of participating sites from 22 to 317, distributed across all seven Emirates.

The UAE has traditionally been a shelter for foreign residents due to its cosmopolitan atmosphere and expat-friendly legislation. Over 200 nationalities have made the UAE their home. The bulk, 88.5% of the people here are expatriates. Indians and Pakistanis are the major expatriate groups, accounting for 27.5 and 12.7% of the overall population, respectively (49). However, according to our findings, around 25.2% of *Pseudomonas* were isolated from Emirati nationals, whereas 5.3 and 4.1% were isolated from Indian and Pakistani experts, respectively. These observed findings should be read with caution because 43.1% of the samples were ascribed to patients whose nationality was not documented in the data, making it unavailable, and whose nationalities were not recognized. With the UAE's expatriate-inclusive and multicultural environment expected to prevail in the coming years, it may be an interesting niche to compare resistance trends in *Pseudomonas* and how these differ by nationality. This approach might shed light on socio-cultural aspects that would be propagating antimicrobial resistance in UAE geographical region (43, 50).

However, because 46% of our samples were from individuals from unknown countries, this study was unable to be performed with our data but remains a fascinating avenue to follow. Furthermore, most patients (54.9%) whose samples were acquired for the study were Abu Dhabi residents. This is consistent with Abu Dhabi being the first Emirate to commence AMR surveillance as well as the Emirate of Abu Dhabi had the highest number of contributing sites, namely 141 (44.2%) out of the total 317 sites enrolled in this study. However, Dubai, not Abu Dhabi, is the most populated Emirate, and samples from Dubai residents accounted for only 23.3% of the sample analyzed in this study.

Approximately 96% of the reported isolates were PA. The remaining proportion was composed of all other species, with *P. putida* and *P. stutzeri* accounting for 1.4% and 0.7%, respectively, while the rest of isolates collected within the study period constituted 0.7%. Of the several species associated with humans, PA can adapt to diverse environments due to a varied array of metabolic pathways and implement pathogenicity owing to the existence of several pathogenicity factors and its high genetic flexibility (2).

PA was more commonly found among inpatients (42.6%, including ICU: 11.5%) than among outpatients (33.6%). Most of the patients were enrolled in medical (20.4%) and surgical (16.4%) departments. In a point prevalence study of 28 European countries from 2016 to 2017, PA was the fifth most prevalent cause of hospital-acquired infections (HAI) (30). *Pseudomonas* has been found in up to 24% of ICU-acquired infections worldwide (31), with resistant PA reaching 48.7% in Brazil (32). A consistent preponderance of adult male patients was observed. Infections caused by PA showed a net reduction in infections in the neonatal and pediatric population since 2016. Though studies show an adult preponderance, PA was more prevalent in children than in adults, in accordance with a recent study (51). In parallel to findings of Sid Ahmed et al. (52), most isolates (26%) came from the respiratory tract, followed by soft tissue samples (ear, 14.0%, pus, 9.2%, and wound, 7.2%), urine (19.5%), and other. Notably, the proportion of outpatient samples was 33.6%, showing that *Pseudomonas* reservoirs are common outside of hospital settings, where they might cause community-acquired pneumonia (53, 54). The real presence of this organism in many environmental places, as well as its transmission to patients in the community is noticeable from our data and warrants additional investigation. PA infections are complicated by this organism's high intrinsic and acquired resistance to several clinically relevant antibiotics, which raises total healthcare costs and may result in severe, life-threatening disease (9–11).

Throughout the study period, PA demonstrated an overall decreasing trend in antibiotic resistance (%R) for several clinically relevant antimicrobials. Carbapenems had the highest resistance among beta-lactam antibiotics (2021: imipenem 14.1%; meropenem 11.4%), while piperacillin/tazobactam, ceftazidime, and cefepime exhibited resistance levels of 7.8, 8.2, and 6.1%, respectively (2021). Studies have reported variable rates of resistance to beta-lactam antibiotics. Resistance to piperacillin-tazobactam and meropenem was equivalent in Western Europe and the United States (23%) but greater in Eastern Europe (34.7%) in patients hospitalized with pneumonia between 2019 and 2021 (33). Meropenem and ceftazidime had the lowest susceptibility (82–83%) in a 5-year retrospective research conducted in Saudi Arabia (39). Earlier studies have revealed 15% carbapenem resistance in PA in Oman (40) and 41% resistance in Lebanon (41). In a retrospective study conducted in Al Ain, UAE from 2004 to 2008, PA demonstrated significant decreases in susceptibility to nearly all antibiotics tested (42). In Dubai, 23.9% of the PA isolates have been reported to be carbapenem-resistant (43). In another study from Saudi Arabia, PA had the highest sensitivity to amikacin (92.6%) and greatest resistance to imipenem (29.5%), ceftazidime (26.1%), meropenem (25.6%), and cefepime (24.3%) (50). In Saudi Arabia, the overall level of cephalosporin resistance in PA was low compared with resistance rates in neighboring countries (96 and 86% in Qatar and Bahrain, respectively) (55). Between 2018 and 2019, PA clinical isolates in Saudi Arabia showed 14.2 and 8.5% resistance to ceftazidime and cefepime, respectively (56). Further, in Mekkah and Jeddah, Saudi Arabia, meropenem showed high resistance (30.6%) as compared to other antibiotics, followed by imipenem (19%). The antibiotics with < 10% resistance were cefepime (8.3%), and piperacillin-tazobactam (4.9%) (57).

In our study, the resistance rate of PA to piperacillin-tazobactam decreased from 9.6% in 2010 to 7.8% in 2021; whereas resistance to ceftazidime and cefepime did not vary much during the study period. Resistance to imipenem increased marginally from 13 to 14%. During the 12 years study period, resistance to imipenem showed a slight increase (from 13 to 14%R), whereas resistance to meropenem showed a decrease (from 14 to 11%R). Considering the period 2017–2021, both carbapenems were showing a decreasing trend of resistance. Among the non-beta-lactam antibiotics, the highest resistance (10.1%) was observed for ciprofloxacin in 2021. Resistance to amikacin, gentamicin, and tobramycin in 2021 was 2.9, 4.2, and 4.2%R, respectively. Our results were in parallel to findings of Hafiz et al. (51) where amikacin was the most effective antibiotic, with susceptibility rates of 92.6%, which is consistent with the findings of previous studies (58, 59). In Qatar, over a three-year study period (2014–2017), the resistance rates to ciprofloxacin, gentamicin, amikacin, and tobramycin were 89.5, 68.0, 54.9, and 52.8%, respectively (52). In our study, PA showed a horizontal trend of resistance for fluoroquinolones (ciprofloxacin) and decreasing trends of resistance for aminoglycosides (gentamicin, amikacin). The rate of MDR decreased from 20.6% in 2020 to 13.1% in 2021. In 2011, the largest percentage of MDR isolates was recorded at 35.6%, which steadily decreased to 13.1% by 2021. Similarly, the highest percentage of XDR isolates was reported in 2010, at 4.9%, while the highest percentage of possible PDR isolates was reported in 2011, at 7.3%. As an overall trend, the percentage of MDR, XDR, and possible PDR isolates generally declined over the study period especially starting from the year 2016, although a slight increase has been observed for %MDR between 2020 and 2021. The slight increase in the %MDR coincides with the COVID-19 pandemic, which is consistent with the trends in the prevalence of other MDR bacteria (50). Every year, MDR PA causes 13–19% of HAIs in the United States (34). In Europe, particularly in Greece, MDR and XDR isolates are prevalent (35).

A retrospective study of adult hospitalized PA patients in Thailand discovered that XDR strains caused 22% of infections, resulting in significantly higher mortality (36). Another prospective study involving 1915 ICU patients in India during 2014–2015 found that MDR and XDR strains caused 47.7% of PA infections (37). Hafiz et al. (51) reported MDR, XDR and PDR rate of 9.7, 11.8, and 0.3% respectively (2019–2021) in Saudi Arabia. MDR isolates increased after 2019, whereas the percentages of XDR and PDR isolates decreased. In Qatar (52), the overall prevalence of MDR PA was 5.6%, which is lower than reports from neighboring countries or regions (55). In many regions, the decline in the prevalence of MDR PA over the study period might be attributed to the efficacy of infection control and antimicrobial stewardship programs (52).

The mortality rate, according to our observations, was about nearly 2.7 times higher in patients associated with CRPA compared with those associated with CSPA. Patients associated with CRPA were 2.3-fold more likely to be admitted to ICU, and their median length of stay was increased by 12 days, as compared to patients associated with CSPA. This is consistent with other findings that indicated a higher mortality rate and poorer clinical outcome in patients with CRPA (60, 61) and highlights the ongoing need for surveillance and control for achieving better health outcomes.

## 5 Conclusion

This 12-year study of the resistance levels and trends in *Pseudomonas* species in the UAE indicated a decline in antimicrobial resistance and in percentages of *Pseudomonas* isolates with MDR and XDR profiles. As the data is suggestive of sustained *Pseudomonas* spp. circulation particularly in the hospital settings, a more stringent implementation of surveillance techniques, infection control strategies, and antibiotic stewardship are suggested to limit the continued dissemination. Further to these findings, continued epidemiological investigation and genetic evolution analysis of *Pseudomonas* are required, to sustain the observed decline in resistance and to provide new strategies for prevention and control. This data also shows that carbapenem-resistant PA are associated with higher mortality, increased ICU admission rates, and a longer hospitalization duration, thus poorer clinical outcome, and higher associated costs. The decreasing trend of multidrug-resistant phenotypes among *Pseudomonas* spp. is encouraging and demonstrates that antimicrobial resistance levels can also decrease over time at national level, however, the underlying reasons for this need to be further studied.

## Data availability statement

The datasets presented in this article are not readily available because the national AMR Surveillance database managed by the UAE Ministry of Health and Prevention (MOHAP) contains confidential health information. Requests to access the datasets should be directed to the UAE Ministry of Health and Prevention (https://mohap.gov.ae/).

## Ethics statement

Ethical approval for this study was provided by the Ministry of Health and Prevention Research Ethics Committee (MOHAP/DXB-REC/J.J.J./No. 86/2023), Dubai Scientific Research Ethics Committee (DSREC-GL17-2023), and Abu Dhabi Health Research and Technology Ethics Committee (DOH/ZHCD/2023/1316). The studies were conducted in accordance with the local legislation and institutional requirements. Written informed consent for participation was not required from the participants or the participants' legal guardians/next of kin in accordance with the national legislation and institutional requirements.

## Author contributions

JT, NA, GM, CM, AS, and DE: conceptualization, data interpretation, and manuscript review and editing. JT, NA, GM, CM, AS, DE, and The UAE AMR Surveillance Consortium: data collection. GM and JT: formal analysis and manuscript preparation. All authors have read and agreed to the published version of the manuscript.

## Supplementary material

Supplementary Figure S1 Length of inpatient hospitalization (days) for CP-susceptible *P. aeruginosa* (CSPA) patients **(A)** versus CP-resistant *P. aeruginosa* (CRPA) patients **(B)** (UAE, 2010-2021).

Supplementary Figure S2 Kaplan-Meyer curve: Probability of still being hospitalized on day x after inpatient admission. CP-susceptible *P. aeruginosa* (CSPA) patients versus CP-resistant *P. aeruginosa* (CRPA) patients (UAE, 2010-2021).

## Appendix

**Table A1:** The UAE AMR Surveillance Consortium.

**Nr**.	**Name**	**Institution**
1	Ahmed Elhag Ahmed	UAE University, College of Medicine and Health Sciences, Al Ain
2	Ahmed F. Yousef	Department of Biology, Center for Membranes and Advanced Water Technology, Khalifa University, Abu Dhabi
3	Amna AlBlooshi	Purelab, Al Ain
4	Dr. Adnan Alatoom	Sheikh Shakhbout Medical City (SSMC), Abu Dhabi
5	Dr. Ahmed Abdulkareem Al Hammadi	Tawam Hospital, Al Ain
6	Dr. Alaa MM Enshasy	Dubai Health Authority, Dubai
7	Dr. Amal Mubarak Madhi	Abu Dhabi Public Health Center, Abu Dhabi
8	Dr. Anju Nabi	Dubai Academic Health Corporation (DAHC), Dubai
9	Dr. Anup Shashikant Poddar	Al Sharq Hospital, Fujairah
10	Dr. Arun Kumar Jha	Danat Al Emarat Hospital, Abu Dhabi
11	Dr. Ayesha Abdulla Al Marzooqi	Abu Dhabi Public Health Center, Abu Dhabi
12	Dr. Bashir Aden	Khalifa University, Abu Dhabi
13	Dr. Deeba Jafri	Purelab, Sheikh Khalifa Medical City, Ajman
14	Dr. Duckjin Hong	Sheikh Khalifa Specialty Hospital (SKSH) RAK
15	Dr. Farah Ibrahim Al-Marzooq	United Arab Emirates University, Al Ain
16	Dr. Fatima Al Dhaheri	United Arab Emirates University, Al Ain
17	Dr. Ghada Abdel Wahab	Abu Dhabi Agriculture and Food Safety Authority, Abu Dhabi
18	Dr. Ghalia Abdul Khader Khoder	University of Sharjah, Sharjah
19	Dr. Gitanjali Avishkar Patil	NMC Specialty Hospital, Abu Dhabi
20	Dr. Hafiz Ahmad	RAK Hospital, Ras Al Khaimah
21	Dr. Hazim Khalifa	Department of Veterinary Medicine, UAE University, Al Ain
22	Dr. Husein Alzabi	Sheikh Khalifa General Hospital, Um al Quwain
23	Dr. Ibrahim Alsayed Mustafa Alhashami	Purelab, Al Qassimi Hospital, Sharjah
24	Dr. Irfaan Akthar	Mediclinic City Hospital, Dubai
25	Dr. Jens Thomsen	Abu Dhabi Public Health Center, Abu Dhabi
26	Dr. John Stelling	WHONET, Boston, USA
27	Dr. Kavita Diddi	Prime Hospital, Dubai
28	Dr. Krishnaprasad Ramabhadran	Burjeel Hospital, Abu Dhabi
29	Dr. Laila Al Dabal	Dubai Academic Health Corporation (DAHC, Dubai)
30	Dr. Madikay Senghore	Khalifa University, Abu Dhabi
31	Dr. Manal Abdel Fattah Ahmed	PureLab, Ras Al Khaimah
32	Dr. Maya Habous	Rashid Hospital, Dubai Academic Health Corporation, Dubai
33	Dr. Moeena Zain	American Hospital Dubai
34	Dr. Monika Maheshwari	Al Zahra Hospital, Dubai
35	Dr. Monika Maheshwari	Medeor 24x7 Hospital, Dubai
36	Dr. Mubarak Saif Alfaresi	Zayed Military Hospital, Abu Dhabi
37	Dr. Mushtaq Khan	United Arab Emirates University, Al Ain
38	Dr. Najiba Abdulrazzaq	Al Kuwait Hospital, Emirates Health Services Establishment, Dubai
39	Dr. Nehad Nabeel Al Shirawi	Al Fujairah Hospital
40	Dr. Nesrin Helmy	Mediclinic Al Noor Hospital - Khalifa Street, Abu Dhabi
41	Dr. Prashant Nasa	NMC Specialty Hospital Al Nahda, Dubai
42	Dr. Rajeshwari T. A. Patil	Burjeel Medical City, Abu Dhabi
43	Dr. Ratna A. Kurahatti	NMC Royal Hospital Khalifa City A, Abu Dhabi
44	Dr. Riyaz Amirali Husain	Dubai Hospital, Dubai Academic Health Corporation, Dubai
45	Dr. Robert Lodu Serafino Wani Swaka	Sheikh Shakhbout Medical City, Abu Dhabi
46	Dr. Savitha Mudalagiriyappa	University Hospital Sharjah, Sharjah
47	Dr. Seema Oommen	Burjeel Medical City, Abu Dhabi
48	Dr. Shaikha Ghannam Alkaabi	Abu Dhabi Public Health Center, Abu Dhabi
49	Dr. Simantini Jog	Fakeeh University Hospital, Dubai
50	Dr. Simantini Jog	King's College Hospital London Dubai Hills, Dubai
51	Dr. Siobhan O‘Sullivan	Khalifa University, Abu Dhabi
52	Dr. Somansu Basu	NMC Specialty Hospital, Al Ain
53	Dr. Yassir Mohammed Eltahir Ali	Animal Wealth Sector, Abu Dhabi Agriculture and Food Safety Authority, Abu Dhabi
54	Dr. Yousuf Mustafa Naqvi	Department of Health Abu Dhabi (DoH), Abu Dhabi
55	Dr. Zulfa Omar Al Deesi	Latifa Maternity & Pediatric Hospital, Dubai
56	Emmanuel Fru Nsutebu	Sheikh Shakhbout Medical City, Abu Dhabi
57	Fouzia Jabeen	Purelab, Sheikh Khalifa Hospital, Abu Dhabi
58	Francis Amirtharaj Selvaraj	Sheikh Khalifa Medical City (SKMC), Abu Dhabi
59	Hadayatullah Ghulam Muhammad	Emirates International Hospital, Al Ain
60	Imene Lazreg	University of Sharjah, Sharjah
61	Kaltham Ali Kayaf	Ministry of Climate Change & Environment (MOCCAE), Dubai
62	Laura Thomsen	University of Freiburg, Germany
63	Leili Chamani-Tabriz	Clemenceau Medical Center, Dubai
64	Pamela Fares Mrad	Abu Dhabi Public Health Center (ADPHC), Abu Dhabi
65	Pascal Frey	Berne University Hospital, Berne, Switzerland
66	Prof. Abiola Senok	College of Medicine, Mohammed Bin Rashid University of Medicine and Health Sciences, Dubai
67	Prof. Agnes-Sonnevend-Pal	University of Pécs, Pécs, Hungary
68	Prof. Andreas Podbielski	University Hospital Rostock, Rostock, Germany
69	Prof. Carole Ayoub Moubareck	College of Natural and Health Sciences, Zayed University, Dubai
70	Prof. Dean Everett	Department of Pathology and Infectious Diseases, College of Medicine, Khalifa University, Abu Dhabi
71	Prof. Godfred A. Menezes	Department of Medical Microbiology and Immunology, RAK Medical and Health Sciences University, Ras Al Khaimah
72	Prof. Hala Ahmed Fouad Ismail	PureLab, Al Qassimi Hospital, Sharjah
73	Prof. Mohamud M. Sheek-Hussein	United Arab Emirates University, Al Ain
74	Prof. Peter Nyasulu	Department of Global Health, Faculty of Medicine and Health Sciences, Stellenbosch University, South Africa
75	Prof. Sameh Soliman	University of Sharjah, Sharjah
76	Prof. Tibor Pal	University of Pécs, Pécs, Hungary
77	Rania El Lababidi	Dept. of Pharmacy Services, Cleveland Clinic Abu Dhabi
78	Saeed Hussein	Erada Center for Treatment and Rehabilitation, Dubai
79	Stefan Weber	Purelab, Abu Dhabi
80	Sura Khamees Majeed	Al Gharbia Hospitals - Madinat Zayed Hospital
81	Syed Irfan Hussein Rizvi	Mediclinic City Hospital, Dubai
82	Timothy Anthony Collyns	Tawam Hospital, Al Ain
83	Zahir Osman Babiker	Sheikh Shakhbout Medical City (SSMC), Abu Dhabi

## References

[1] Behzadi P Ambrosi C Scribano D Zanetti S Sarshar M Gajdács M. Editorial: current perspectives on *Pseudomonas aeruginosa*: epidemiology, virulence and contemporary strategies to combat multidrug-resistant (MDR) pathogens. Front Microbiol. (2022) 13:975616. 10.3389/fmicb.2022.975616 35958138 PMC9363151

[2] Rumbaugh KP. Genomic complexity and plasticity ensure Pseudomonas success. FEMS Microbiol Lett. (2014) 356:141–3. 10.1111/1574-6968.12517 25060810

[3] Zhu NJ Rawson TM Mookerjee S Price JR Davies F Otter J. Changing patterns of bloodstream infections in the community and acute care across 2 coronavirus disease 2019 epidemic waves: a retrospective analysis using data linkage. Clin Infect Dis. (2022) 75:e1082–91. 10.1093/cid/ciab869 34596212 PMC9402624

[4] Ng QX Ong NY Lee DYX Yau CE Lim YL Kwa ALH. Trends in *Pseudomonas aeruginosa* (*P. aeruginosa*) Bacteremia during the COVID-19 pandemic: a systematic review. Antibiotics. (2023) 12:409. 10.3390/antibiotics12020409 36830319 PMC9952731

[5] Engel JN. Molecular pathogenesis of acute *Pseudomonas aeruginosa* infections. In:Hauser A Rello J, editors. Severe Infections Caused by Pseudomonas aeruginosa. New York, NY: Kluwer Academic/Plenum Press (2003). p. 201–30.

[6] Matos ECOD Andriolo RB Rodrigues YC Lima PDLD Carneiro ICDRS Lima KVB. Mortality in patients with multidrug-resistant Pseudomonas aeruginosa infections: a meta-analysis. Rev Soc Bras Med Trop. (2018) 51:415–20. 10.1590/0037-8682-0506-2017 30133622

[7] Wood SJ Kuzel T. M Shafikhani SH. *Pseudomonas aeruginosa*: infections, animal modeling, and therapeutics. Cells. (2023) 12:199. 10.3390/cells12010199 36611992 PMC9818774

[8] Kadri SS Adjemian J Lai YL Spaulding AB Ricotta E Prevots DR. National Institutes of Health Antimicrobial Resistance Outcomes Research Initiative (NIH-ARORI). Difficult-to-Treat Resistance in Gram-negative Bacteremia at 173 US Hospitals: retrospective cohort analysis of prevalence, predictors, and outcome of resistance to all first-line agents. Clin Infect Dis. (2018) 67:1803–14. 10.1093/cid/ciy378 30052813 PMC6260171

[9] Pang Z Raudonis R Glick BR Lin TJ Cheng Z. Antibiotic resistance in *Pseudomonas aeruginosa*: mechanisms and alternative therapeutic strategies. Biotechnol Adv. (2019) 37:177–92. 10.1016/j.biotechadv.2018.11.013 30500353

[10] Chen Z. Mechanisms and clinical relevance of *Pseudomonas aeruginosa* Heteroresistance. Surg Infect. (2023) 24:27–38. 10.1089/sur.2022.349 36622941

[11] Verdial C Serrano I Tavares L Gil S Oliveira M. Mechanisms of antibiotic and biocide resistance that contribute to *Pseudomonas aeruginosa* persistence in the hospital environment. Biomedicines. (2023) 11:1221. 10.3390/biomedicines11041221 37189839 PMC10135826

[12] Magiorakos AP Srinivasan A Carey RB Carmeli Y Falagas ME Giske CG. Multidrug-resistant, extensively drug-resistant and pandrug-resistant bacteria: an international expert proposal for interim standard definitions for acquired resistance. Clin Microbiol Infect. (2012) 18:268–81. 10.1111/j.1469-0691.2011.03570.x 21793988

[13] Shrivastava SR Shrivastava PS Ramasamy J. World health organization releases global priority list of antibiotic-resistant bacteria to guide research, discovery, and development of new antibiotics. J Med Soc. (2018) 32:76–7. 10.4103/jms.jms_25_17

[14] Centers for Disease Control Prevention. Antibiotic Resistance Threats in the United States. (2019). Available from: https://www.cdc.gov/drugresistance/pdf/threats-report/2019-ar-threats-report-508.pd (accessed June 18, 2023).

[15] Quinn PJ Markey BK Leonard FC FitzPatrick ES Fanning S Hartigan PJ. Antibacterial resistance. In: Veterinary Microbiology and Microbial Disease. Chichester: Wiley-Blackwell (2011).

[16] Boerlin P White DG. Antimicrobial resistance and its epidemiology. In:Giguère S Prescott JF Dowling PM, editors. Antimicrobial Therapy in Veterinary Medicine. Ames, IA: Wiley-Blackwell (2013). p. 21–40.

[17] Moradali MF Ghods S Rehm BHA. *Pseudomonas aeruginosa* lifestyle: a paradigm for adaptation, survival, and persistence. Front Cell Infect Microbiol. (2017) 7:39. 10.3389/fcimb.2017.00039 28261568 PMC5310132

[18] Mohr KI. History of antibiotics research. Curr Top Microbiol Immunol. (2016) 398:237–72. 10.1007/82_2016_499 27738915

[19] Munita JM Arias CA. Mechanisms of antibiotic resistance. Microbiol Spectr. (2016) 4. 10.1128/microbiolspec.VMBF-0016-2015 PMC4888801 27227291

[20] Llanes C Köhler T Patry I Dehecq B van Delden C Plésiat P. Role of the MexEF-OprN efflux system in low-level resistance of *Pseudomonas aeruginosa* to ciprofloxacin. Antimicrob Agents Chemother. (2011) 55:5676–84. 10.1128/AAC.00101-11 21911574 PMC3232816

[21] Llanes C Pourcel C Richardot C Plésiat P Fichant G Cavallo JD. Diversity of β-lactam resistance mechanisms in cystic fibrosis isolates of *Pseudomonas aeruginosa*: a French multicentre study. J Antimicrob Chemother. (2013) 68:1763–71. 10.1093/jac/dkt115 23629014

[22] Wolter DJ Lister PD. Mechanisms of β-lactam resistance among Pseudomonas aeruginosa. Curr Pharm Des. (2013) 19:209–22. 10.2174/138161213804070311 22894618

[23] Majiduddin FK Materon IC Palzkill TG. Molecular analysis of beta-lactamase structure and function. Int J Med Microbiol. (2002) 292:127–37. 10.1078/1438-4221-00198 12195735

[24] Hagel S Makarewicz O Hartung A Weiß D Stein C Brandt C. ESBL colonization and acquisition in a hospital population: The molecular epidemiology and transmission of resistance genes. PLoS ONE. (2019) 14:e0208505. 10.1371/journal.pone.0208505 30640915 PMC6331103

[25] Lemarié C Legeay C Lasocki S Mahieu R Kouatchet A Bahier L. Extended-spectrum β-lactamase Enterobacteriaceae (ESBLE) in intensive care units: strong correlation with the ESBLE colonization pressure in patients but not same species. J Hosp Infect. (2020) 104:53–6. 10.1016/j.jhin.2019.08.007 31408692

[26] Lynch JP 3rd Zhanel GG. *Pseudomonas aeruginosa* pneumonia: evolution of antimicrobial resistance and implications for therapy. Semin Respir Crit Care Med. (2022) 43:191–218. 10.1055/s-0041-1740109 35062038

[27] Kariminik A Baseri-Salehi M Kheirkhah B. *Pseudomonas aeruginosa* quorum sensing modulates immune responses: an updated review article. Immunol Lett. (2017) 190:1–6. 10.1016/j.imlet.2017.07.002 28698104

[28] Jurado-Martín I Sainz-Mejías M McClean S. *Pseudomonas aeruginosa*: an audacious pathogen with an adaptable arsenal of virulence factors. Int J Mol Sci. (2021) 22:3128. 10.3390/ijms22063128 33803907 PMC8003266

[29] Virieux-Petit M Hammer-Dedet F Aujoulat F Jumas-Bilak E Romano-Bertrand S. From copper tolerance to resistance in *Pseudomonas aeruginosa* towards patho-adaptation and hospital success. Genes. (2022) 13:301. 10.3390/genes13020301 35205346 PMC8872213

[30] Suetens C Latour K Kärki T Ricchizzi E Kinross P Moro ML. Prevalence of healthcare-associated infections, estimated incidence and composite antimicrobial resistance index in acute care hospitals and long-term care facilities: results from two European point prevalence surveys, 2016 to 2017. Euro Surveill. (2018) 23:1800516. 10.2807/1560-7917.ES.2018.23.46.1800516 30458912 PMC6247459

[31] Vincent JL Sakr Y Singer M Martin-Loeches I Machado FR Marshall JC. Prevalence and outcomes of infection among patients in intensive care units in 2017. JAMA. (2020) 323:1478–87. 10.1001/jama.2020.2717 32207816 PMC7093816

[32] Ribeiro ÁCDS Crozatti MTL Silva AAD Macedo RS Machado AMO Silva ATA. Pseudomonas aeruginosa in the ICU: prevalence, resistance profile, and antimicrobial consumption. Rev Soc Bras Med Trop. (2019) 53:e20180498. 10.1590/0037-8682-0498-2018 31859938 PMC7083346

[33] Losito AR Raffaelli F Del Giacomo P Tumbarello M. New Drugs for the treatment of pseudomonas aeruginosa infections with limited treatment options: a narrative review. Antibiotics. (2022) 11:579. 10.3390/antibiotics11050579 35625223 PMC9137685

[34] Raman G Avendano EE Chan J Merchant S Puzniak L. Risk factors for hospitalized patients with resistant or multidrug-resistant *Pseudomonas aeruginosa* infections: a systematic review and meta-analysis. Antimicrob Resist Infect Control. (2018) 7:79. 10.1186/s13756-018-0370-9 29997889 PMC6032536

[35] Pérez A Gato E Pérez-Llarena J Fernández-Cuenca F Gude MJ Oviaño M. High incidence of MDR and XDR *Pseudomonas aeruginosa* isolates obtained from patients with ventilator-associated pneumonia in Greece, Italy and Spain as part of the MagicBullet clinical trial. J Antimicrob Chemother. (2019) 74:1244–52. 10.1093/jac/dkz030 30753505

[36] Palavutitotai N Jitmuang A Tongsai S Kiratisin P Angkasekwinai N. Epidemiology and risk factors of extensively drug-resistant *Pseudomonas aeruginosa* infections. PLoS ONE. (2018) 13:e0193431. 10.1371/journal.pone.0193431 29470531 PMC5823452

[37] Gill JS Arora S Khanna SP Kumar KH. Prevalence of multidrug-resistant, extensively drug-resistant, and pandrug-resistant *Pseudomonas aeruginosa* from a tertiary level intensive care unit. J Glob Infect Dis. (2016) 8:155–9. 10.4103/0974-777X.192962 27942195 PMC5126754

[38] Ibrahim ME. High antimicrobial resistant rates among Gram-negative pathogens in intensive care units. A retrospective study at a tertiary care hospital in Southwest Saudi Arabia. Saudi Med J. (2018) 39:1035–43. 10.15537/smj.2018.10.22944 30284588 PMC6201019

[39] Alhumaid S Al Mutair A Al Alawi Z Alzahrani AJ Tobaiqy M Alresasi AM. Antimicrobial susceptibility of gram-positive and gram-negative bacteria: a 5-year retrospective analysis at a multi-hospital healthcare system in Saudi Arabia. Ann Clin Microbiol Antimicrob. (2021) 20:43. 10.1186/s12941-021-00450-x 34118930 PMC8196925

[40] Al-Yaqoubi M Elhag K. Susceptibilities of common bacterial isolates from Oman to old and new antibiotics. Oman Med J. (2008) 23:173–8. 22359709 PMC3282317

[41] Hammoudi HD Moubareck CA Sarkis DK. Heterogeneity of carbapenem resistance mechanisms among gram-negative pathogens in Lebanon: results of the first cross-sectional countrywide study. Microb Drug Resist. (2017) 23:733–43. 10.1089/mdr.2016.0077 28080212

[42] Al-Kaabi MR Tariq WU Hassanein AA. Rising bacterial resistance to common antibiotics in Al Ain, United Arab Emirates. East Mediterr Health J. (2011) 17:479–84. 10.26719/2011.17.6.479 21796964

[43] Moubareck CA Halat DH Akkawi C Nabi A AlSharhan MA AlDeesi ZO. Role of outer membrane permeability, efflux mechanism, and carbapenemases in carbapenem-nonsusceptible Pseudomonas aeruginosa from Dubai hospitals: results of the first cross-sectional survey. Int J Infect Dis. (2019) 84:143–50. 10.1016/j.ijid.2019.04.027 31204002

[44] Karruli A Catalini C D'Amore C Foglia F Mari F Harxhi A. Evidence-based treatment of *Pseudomonas aeruginosa* infections: a critical reappraisal. Antibiotics. (2023) 12:399. 10.3390/antibiotics12020399 36830309 PMC9952410

[45] Thomsen J Senok A Alatoom A Nabi A Aden B Moubareck CA. United Arab Emirates Surveillance of Antimicrobial Resistance Annual Report, 2022. The Ministry of Health Prevention; National Sub-Committee for AMR Surveillance (2022). Available from: https://mohap.gov.ae/assets/f5a5705/National%20AMR%20Surveillance%20Report%202022%20MOHAP_638205230312192483.pdf.aspx (accessed June 20, 2023).

[46] Thomsen J Abdulrazzaq NM AlRand H The UAE AMR Surveillance Consortium. Surveillance of antimicrobial resistance in the United Arab Emirates: the early implementation phase. Front Public Health. (2023) 11:1247627. 10.3389/fpubh.2023.1247627 38074700 PMC10704098

[47] Clinical Laboratory and Standards Instiute (CLSI). Performance Standards for Antimicrobial Susceptibility Testing. 30th ed. Wayne, PA (2020).

[48] Micek ST Wunderink RG Kollef MH Chen C Rello J Chastre J. An international multicenter retrospective study of *Pseudomonas aeruginosa* nosocomial pneumonia: impact of multidrug resistance. Crit Care. (2015) 19:219. 10.1186/s13054-015-0926-5 25944081 PMC4446947

[49] United Arab Emirates Population Statistics. GMI Blogger (2023). Available online at: https://www.globalmediainsight.com/blog/uae-population-statistics/ (accessed June 18, 2023).

[50] Zowawi HM Syrmis MW Kidd TJ Balkhy HH Walsh TR Al Johani SM. Identification of carbapenem-resistant *Pseudomonas aeruginosa* in selected hospitals of the Gulf Cooperation Council States: dominance of high-risk clones in the region. J Med Microbiol. (2018) 67:846–53. 10.1099/jmm.0.000730 29664716

[51] Hafiz TA Bin Essa EA Alharbi SR Alyami AS Alkudmani ZS Mubaraki MA. Epidemiological, microbiological, and clinical characteristics of multi-resistant *Pseudomonas aeruginosa* isolates in King Fahad Medical City, Riyadh, Saudi Arabia. Trop Med Infect Dis. (2023) 8:205. 10.3390/tropicalmed8040205 37104331 PMC10145365

[52] Sid Ahmed MA Abdel Hadi H Abu Jarir S Ahmad Khan F Arbab MA Hamid JM. Prevalence and microbiological and genetic characteristics of multidrug-resistant Pseudomonas aeruginosa over three years in Qatar. Antimicrob Steward Healthc Epidemiol. (2022) 2:e96. 10.1017/ash.2022.226 36483382 PMC9726487

[53] Woods EC Cohen GM Bressman E Lin D Zeitouni NE Beckford C. Community-acquired cavitary Pseudomonas pneumonia linked to use of a home humidifier. Case Rep Infect Dis. (2017) 2017:5474916. 10.1155/2017/5474916 29527364 PMC5763093

[54] Lutz JK Lee J. Prevalence and antimicrobial-resistance of *Pseudomonas aeruginosa* in swimming pools and hot tubs. Int J Environ Res Public Health. (2011) 8:554–64. 10.3390/ijerph8020554 21556203 PMC3084478

[55] Al-Orphaly M Hadi HA Eltayeb FK Al-Hail H Samuel BG Sultan AA. Epidemiology of multidrug-resistant *Pseudomonas aeruginosa* in the Middle East and North Africa Region. mSphere. (2021) 6:e00202–21. 10.1128/mSphere.00202-21 34011686 PMC8265635

[56] Doumith M Alhassinah S Alswaji A Alzayer M Alrashidi E Okdah L. Genomic characterization of carbapenem-non-susceptible *Pseudomonas aeruginosa* clinical isolates from Saudi Arabia revealed a global dissemination of GES-5-producing ST235 and VIM-2-producing ST233 sub-lineages. Front Microbiol. (2022) 12:765113. 10.3389/fmicb.2021.765113 35069471 PMC8770977

[57] Khan MA Faiz A. Antimicrobial resistance patterns of *Pseudomonas aeruginosa* in tertiary care hospitals of Makkah and Jeddah. Ann Saudi Med. (2016) 36:23–8. 10.5144/0256-4947.2016.23 26922684 PMC6074268

[58] Galani I Papoutsaki V Karantani I Karaiskos I Galani L Adamou P. *In vitro* activity of ceftolozane/tazobactam alone and in combination with amikacin against MDR/XDR *Pseudomonas aeruginosa* isolates from Greece. J Antimicrob Chemother. (2020) 75:2164–72. 10.1093/jac/dkaa160 32449909

[59] Alnasser AHA Al-Tawfiq JA Ahmed HAA Alqithami SMH Alhaddad ZMA Rabiah ASM. Public knowledge, attitude and practice towards antibiotics use and antimicrobial resistance in Saudi Arabia: a web-based cross-sectional survey. J Public Health Res. (2021) 10:2276. 10.4081/jphr.2021.2276 34313091 PMC8715265

[60] Lodise TP Bassetti M Ferrer R Naas T Niki Y Paterson DL. All-cause mortality rates in adults with carbapenem-resistant gram-negative bacterial infections: a comprehensive review of pathogen-focused, prospective, randomized, interventional clinical studies. Expert Rev Anti Infect Ther. (2021) 12:1–13. 10.1080/14787210.2022.2020099 34937518

[61] Qin J Zou C Tao J Wei T Yan L Zhang Y. Carbapenem resistant *Pseudomonas aeruginosa* infections in elderly patients: antimicrobial resistance profiles, risk factors and impact on clinical outcomes. Infect Drug Resist. (2022) 15:2301–14. 10.2147/IDR.S358778 35517901 PMC9064054

