# Supplementary figures and images for: Evolving trends among Pseudomonas aeruginosa: a 12-year retrospective study from the United Arab Emirates
Source: Front Public Health. 2023 Nov 30;11:1243973. doi: 10.3389/fpubh.2023.1243973 (PMC10721971; doi:10.3389/fpubh.2023.1243973)

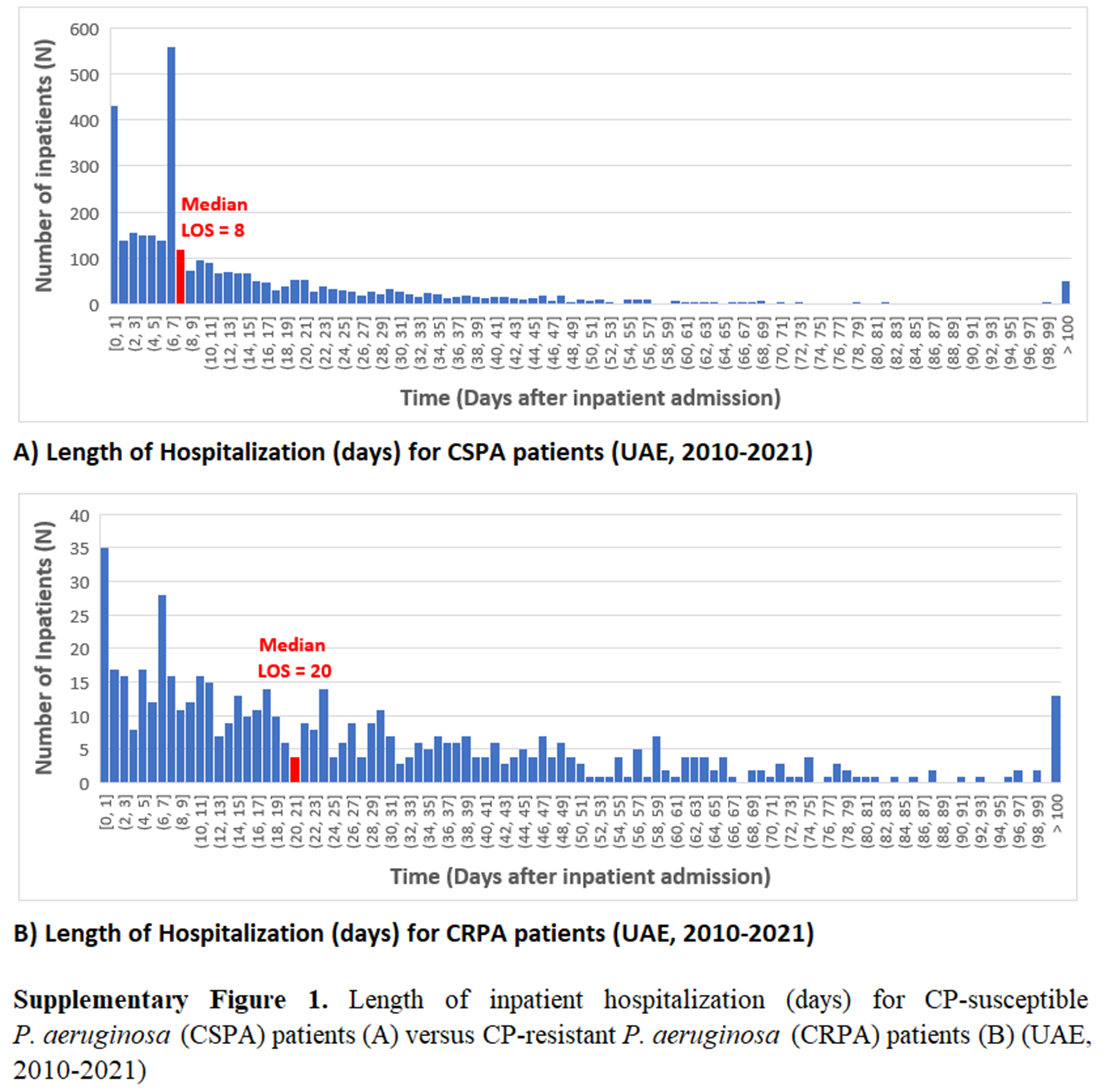

Supplement: Supplementary Figure S1 — Length of inpatient hospitalization (days) for CP-susceptible P. aeruginosa (CSPA) patients (A) versus CP-resistant P. aeruginosa (CRPA) patients (B) (UAE, 2010-2021). [file Image_1.TIF]

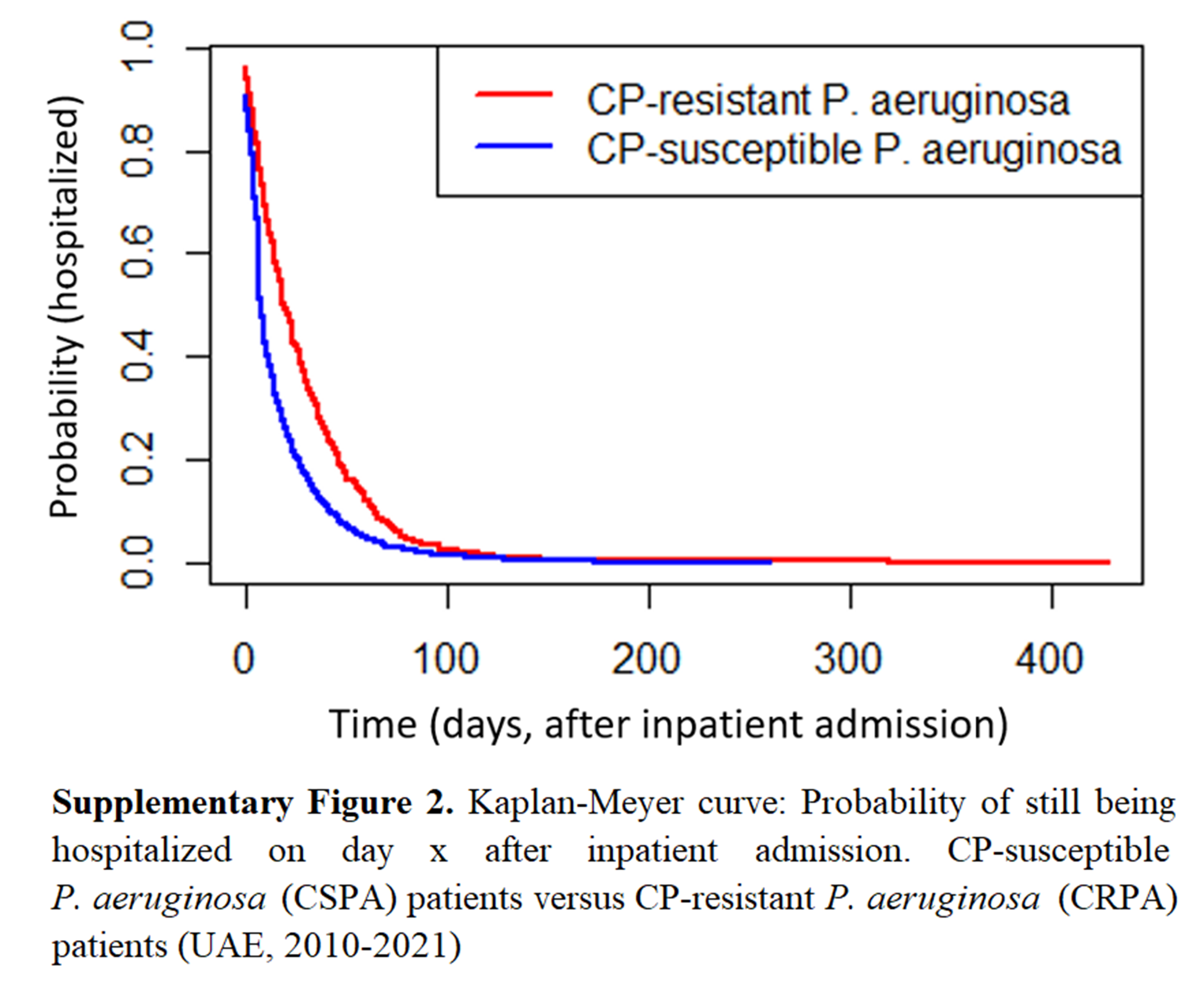

Supplement: Supplementary Figure S2 — Kaplan-Meyer curve: Probability of still being hospitalized on day x after inpatient admission. CP-susceptible P. aeruginosa (CSPA) patients versus CP-resistant P. aeruginosa (CRPA) patients (UAE, 2010-2021). [file Image_2.TIF]
